# Barriers to attendance at a tertiary hospital’s perinatal mortality meeting

**DOI:** 10.1007/s11845-022-03137-0

**Published:** 2022-09-02

**Authors:** Barbara Burke, Sophie Boyd, Karen McNamara, Keelin O’Donoghue

**Affiliations:** 1 Pregnancy Loss Research Group, Department of Obstetrics and Gynaecology, Cork University Maternity Hospital, University College Cork, Wilton, Cork Ireland; 2 The Irish Centre for Maternal and Child Health Research (INFANT), University College Cork, Cork, Ireland

**Keywords:** Clinical governance, Multi-disciplinary team meeting, Perinatal death, Perinatal mortality

## Abstract

**Background:**

Perinatal mortality multi-disciplinary team meetings (PM-MDTMs) offer a forum for multi-disciplinary discussion of poor perinatal outcomes. They ensure a thorough understanding of individual cases and present an important learning opportunity for healthcare professionals (HCPs). Attendance at PM-MDTMs in this tertiary maternity hospital has been low.

**Aims:**

We aimed to identify barriers which may be targeted to improve attendance and engagement.

**Methods:**

An anonymous questionnaire was developed, and all HCPs invited to participate. Demographic data on respondents was collected, as was knowledge of PM-MDTMs, their purpose and relevance to clinical practice, and barriers to attendance at meetings. A total of 78 responses were obtained and analysed.

**Results:**

Self-reported understanding of the purpose and format PM-MDTMs was high (84.6% (66/78) and 65.4% (51/78), respectively), while only 50% (39/78) of respondents provided an accurate description of either. Only 50% (39/78) reported having attended a meeting in the hospital, of whom 61.5% (24/39) described the correct meeting. Of these, 37.5% (9/24) reported attending regularly and 70.8% (17/24) found the meeting relevant to their clinical practice. Of the 33.33% (26/78) who reported attending a PM-MDTM in another hospital, 73.1% (19/26) accurately described the meeting, 63.1% (12/19) of these attended regularly, and 100% (19/19) found it relevant. Three main qualitative themes emerged as barriers to attendance and were areas for suggested improvements: workload and staffing levels, meeting logistics, and lack of communication and education regarding PM-MDTMs.

**Conclusions:**

Communication regarding PM-MDTMs and their learning opportunities needs to improve. Lack of engagement is likely compounded by high workloads and staffing levels, but these issues should be surmountable.

**Supplementary Information:**

The online version contains supplementary material available at 10.1007/s11845-022-03137-0.

## Introduction

Perinatal mortality is a devastating outcome for both families and healthcare providers [1, 2]. The WHO estimates that there are 5.3 million perinatal deaths annually worldwide, which are due to a variety of modifiable and non-modifiable factors [3]. It is recognised in the literature that review of perinatal deaths is essential to the continuous improvement of clinical care [4]. This can be done in a variety of ways, including national audit, confidential enquiries, and local reviews. Local reviews are associated with a lower cost and simpler organisational structure compared to other options, and in many regions feed into national reviews [5, 6]. National reviews in both Ireland and the UK provide tools to support quality standardised assessment of cases at a local level [6].

Local reviews generally take the form of multi-disciplinary team meetings (i.e. perinatal mortality multi-disciplinary team meetings/PM-MDTMs) and include input from obstetrics, midwifery, neonatology, and pathology [5]. The aims of morbidity and mortality reviews are to improve patient safety, improve quality of care, and act as a learning resource, all without the apportioning of blame [7, 8]. Formal guidance on the execution of such reviews aims to ensure processes which provide a thorough understanding of individual cases, and allow for appropriate follow-up, as well as presenting an important learning opportunity for all healthcare professionals (HCPs) [e.g. 9, 10]. For the purposes of this study, and based on work by Helps et al. [5], we defined a perinatal mortality MDT meeting as a formal collaborative meeting between specialities, including obstetrics, midwifery, neonatology, and pathology, which allows the discussion of cases, seeking to identify factors contributing to perinatal mortality and improve care without apportioning blame.

This study was conducted in a large maternity hospital in the Republic of Ireland, which is the tertiary referral centre for the Ireland South Maternity Directorate, and is a teaching hospital affiliated with University College Cork and the RCPI Obstetrics & Gynaecology training schemes. The hospital has had an approximately bi-monthly PM-MDTM, held for 2 h on a Friday afternoon within the hospital. A previous audit of PM-MDTMs within the hospital showed consistently low attendance [11]; from 2013 to 2016, a median of 8 meetings were held per year, at which an average of 14 staff attended per meeting. This represents approximately 3% of the total workforce of 432 staff members attending per meeting in 2016 [12]. We aimed to identify potential barriers to attendance that may be targeted to facilitate improved attendance and engagement with the PM-MDT meeting.

## Methods

The sign in sheets of PM-MDT meetings in the hospital from 2017 to 2020 were reviewed in order to determine levels of attendance across different professions.

An anonymous questionnaire (see Appendix 1), containing both open and closed questions, was developed. Most questions required simple yes or no answers, but some included room for free text responses. Demographic data on respondents was collected, including current occupation and length of service, both at the hospital and generally in the maternity services. Further questions assessed respondents’ knowledge of PM-MDTMs, their format and purpose, and their experience of PM-MDTMs both within this hospital and at others. In addition, we asked respondents to detail barriers to attendance within the hospital, as well as to provide suggestions for potential improvements.

To get a broad assessment of the HCPs within the tertiary maternity hospital, all staff who had direct clinical patient contact within the unit were invited to participate in the study. This included medical staff, midwifery and nursing staff, healthcare assistants, and allied health professionals. The questionnaire was available both electronically and in hard copy throughout all clinical areas in the hospital. Staff were invited to participate by the authors or their line managers at ward/staff meetings, and sealed questionnaire collection boxes that advertised the study were left in prominent sites in staff-only areas of each ward.

Ethical approval was sought for this project from the Clinical Research Ethics Committee of the Cork Teaching Hospitals (Ref. No: ECM 4 (cc) 14/01/2020), and consent for participation in the study was assumed from the submission of a completed questionnaire.

Descriptive statistics were performed using IBM SPSS software. Qualitative analysis was performed on free text responses to open questions. A grounded theory/open coding approach was chosen to examine responses for underlying meaning and similarity [13, 14]. This method was used as data was from questionnaire responses only, and no interviews were conducted. A list of initial codes was constructed from analysis of individual questionnaire responses. These codes were divided into themes and analysed. Initial analysis was performed by the primary author, and reviewed by one of the co-authors.

## Results

### Attendance at PM-MDT meetings 2017–2020

Records were available for were 24 PM-MDTMs from 2017 to 2020 (median 6 per year, range 4–8). The median attendance for these meetings was 9.5 (range 4–24). Of note, there were higher attendance rates in 2020, which saw a switch to online meetings secondary to COVID-19 pandemic restrictions (median 22, range 8–24).

Attendees included consultant obstetricians, neonatologists, and pathologists, as well as obstetric and neonatal doctors in training, bereavement and loss (B&L) midwife specialists and quality and patient safety (QPS) management (Table 1). No other midwifery management or hospital management representatives, nor administration staff, attended meetings, nor did any staff midwives. Twelve obstetric consultants attended the meetings on at least one occasion, 5 of whom only attended once.

**Table 1:** PM-MDTM attendees by professional group, and hospital employment rates

	% Meetings at which represented	Median attendance per meeting	Numbers employed, 2019 [15]
Consultants			22
Obstetrics	100%	3	
Neonatology	50%	0.5	
Pathology	100%	1	
Doctors in training			31
Obstetrics	88%	3	
Neonatology	38%	0	
Midwifery			419
Management, QPS	63%	1	
Management, other	0%	0	
B&L	83%	1	
Other	0%	0	

### Questionnaire responses – quantitative analysis

A total of 78 responses were received, representing a wide range of HCPs. Respondents included 6 consultants, 26 doctors in training, 42 midwives, 3 nurses, and 1 healthcare assistant; no responses were returned from allied health professionals. This represents 16.5% (78/472) of the total hospital workforce in 2019 [15]. Of these, 43.6% (34/78) of participants had worked in the maternity services for over 10 years, while 34.6% (27/78) had worked at this hospital for less than 1 year. The basic demographics of respondents are summarised in Table 2.

**Table 2:** Demographics of participants

Characteristic	Number	%
**Position**		
Consultant	6	7.7%
Doctor in training	26	33.3%
Midwifery/nursing	45	57.7%
Healthcare assistant	1	1.3%
**Sub-speciality/grade**		
**Obstetrics**	27	
Consultant	3	
Senior/specialist registrar	9	
Junior registrar/registrar	8	
Senior house officer	7	
**Neonatology**	5	
Consultant	3	
Senior/specialist registrar	2	
**Midwifery/nursing**	45	
Manager	7	
Specialist	1	
Registered midwife	34	
Registered nurse	3	
**Years working in maternity services**		
> 10 years	34	43.6%
5–10 years	18	23.1%
< 5 years	17	21.8%
< 1 year	9	11.5%
**Years working at this hospital**		
> 10 years	29	37.2%
5–10 years	5	6.4%
< 5 years	17	21.8%
< 1 year	27	34.6%

Self-reported understanding of the purpose and format of a PM-MDT meeting was high: 84.6% (66/78) and 65.4% (51/78), respectively. However, when asked to describe both, only 50% (39/78) of respondents provided an accurate description. Of the respondents, 32.1% (25/78) detailed either a clearly different meeting or gave a description that could apply to any meeting within the hospital, with no identifiable traits of a PM-MDTM. The group who was least likely to provide an accurate description was registered midwives, of whom only 32.4% (11/34) described a PM-MDTM correctly. This was significantly lower than other respondent groups (*χ*^2^ 8.6371, *p* = 0.0345). The majority (85.9%, 67/78) reported that they were aware of a PM-MDTM within the hospital, but fewer were aware of its frequency (46.2%, 36/78) or location (57.7%, 45/78).

Only 50% (39/78) reported having attended a PM-MDTM at this hospital, of whom 61.5% (24/39) described the correct meeting. Of these, 37.5% (9/24) reported attending the meeting regularly and 70.8% (17/24) found it relevant to their clinical practice. In contrast, of those who reported attending PM-MDTMs in other hospitals (33.33%, 26/78), 73.1% (19/26) described the correct meeting type. Of these, 63.1% (12/19) reported attended meetings regularly, and 100% (19/19) found them relevant to their practice.

### Questionnaire responses – qualitative analysis

Qualitative analysis was conducted only for completed responses where an accurate description of a PM-MDTM was included. Three main themes were identified from the data and were consistently cited both as barriers to attendance (*n* = 37, Table 3) and as areas to target in improving the meeting and its attendance (*n* = 34, Table 4). These were staffing levels and workload, meeting logistics, and a lack of communication and education regarding PM-MDTMs. Only one respondent, a consultant obstetrician, felt that there were no barriers to attendance of the meeting.

**Table 3:** Barriers to attendance at PM-MDT meeting, by theme

Theme	Participant quotes
Staffing levels and workload	“Lack of staff… Not allocated to time off to attend” — Midwife
	“Clinical activities elsewhere - understaffing means NCHD [Non Consultant Hospital Doctor] staff are stretched thin across the service” — Doctor in Training
	“Same amount of work to do when they return to ward. Same caseload.” — Midwife Manager
Meeting logistics	“Day/time its on, length of meeting (2 h), clinical staff unable to be released for this long” — Midwife Manager
	“For neonates – clashes with paeds radiology [meeting]” — Doctor in Training
Lack of communication and education	“Not understanding what is / the benefits of them” — Specialist Midwife
	“Perceptions about what many are about” — Midwife Manager
	“Perceived to be open ‘to the doctors’.” — Midwife
	“Not informed / invited…. Not aware its open to anyone to attend.” — Midwife
	“Lack of consultant interest (selective)” — Doctor in Training

**Table 4:** Suggested improvements for the PM-MDT meeting and its attendance, by theme

Theme	Participant quotes
Staffing levels and workload	“Different areas to attend meeting ‘by invitation’, especially staff involved in cases” — Midwife Manager
	“Need a representative from all areas, especially if involved in cases being discussed. Rep could feed back information. Perhaps advise teams of cases relevant to them for learning.” — Midwife Manager
Meeting logistics	“Option to attend online” — Midwife
	“Change time of meeting to morning if possible, if remains on Friday. If alternative day/afternoon/evening would be more accessible.” — Midwife Manager
	“Midweek lunchtime meeting” — Doctor in Training
Lack of communication and education	“Promote more - visually - posters on wards etc. Present benefits at grand rounds.” — Specialist Midwife
	“Ward CMMs need to be on board to actively send staff / prioritise attendance” — Midwife
	“Encouraging NCHDs to attend, more consultant attendance.” — Doctor in Training

#### Theme one: staffing levels and workload

The current low staffing levels and resultant high workload in the hospital was reported to prevent staff from being released from clinical duties to attend the meeting. This was cited by members of all staff groups and was the most commonly reported barrier, appearing in 75.6% (28/37) of responses. This was also the most common theme for suggested improvements, with 44.1% (15/34) suggesting potential changes. These included actively facilitating staff to go as part of their rostered duties. In particular, it was felt by management that specific staff involved in cases should be facilitated to attend, and could then feedback learning points to their respective clinical areas.

#### Theme two: meeting logistics

The current timing and duration of the meeting was cited as a barrier by 27.02% (10/37) of respondents, again spread throughout all staff groups. This ranged from general comments to specific concerns or clashes. Various suggestions were offered to improve meeting logistics by 32.4% (11/34) of respondents. The majority felt a midweek lunchtime meeting would be more highly attended. It was suggested by one participant that online meetings may be more successful.

#### Theme three: lack of communication and education

Lack of knowledge regarding PM-MDTMs and communication regarding their being held in the hospital was cited by 21.6% (8/37) of respondents. In contrast to the previous themes, this was reported primarily by midwifery staff (75%, 6/8). A lack of understanding of the meeting’s contents was reported as a barrier to attendance. Some respondents were aware of the meetings but did not feel that they could attend, citing a perception of the meeting as a doctors-only event. Improved education and communication were cited as an area for improvement by 29.4% (10/34). Explicit communication of the meetings’ existence and logistics was suggested as a method to boost attendance. Suggestions for conveying this information ranged from announcements at ward handover meetings to posting on notice boards or ward WhatsApp groups. It was also suggested that formal education on the benefits of PM-MDTMs might aid improvement of the meeting. Implicit communication regarding the meeting and its importance was also cited as a barrier and area for improvement. A lack of attendance or importance placed on the meeting by some consultants and midwifery management was noted by respondents under their clinical leadership.

## Discussion

### Main findings

This study confirmed low attendance at PM-MDTMs at the hospital, and the attendance appears to have dropped from the previous audit findings. The improvement in median number of attendees following the switch to online meetings is interesting and provides one potential route to improve staff engagement. The lower engagement of neonatology staff is possibly a result of two formal meetings clashing within the hospital.

Of those who reported attending PM-MDTMs, the majority found it beneficial to their clinical practice. This was true both in this hospital and where they had attended elsewhere. Staff who had attended these meetings in other hospital were more likely to describe the correct meeting type and were twice as likely to have attended them regularly. This potentially reflects different approaches to education and advertising of the meeting.

A relatively large number of staff had worked within the maternity services for over 10 years; however, many had also only worked at this hospital for less than 1 year. This potentially reflects a large turnover of staff, new members of which may have no knowledge of the meeting. This cohort may be an easy target for education regarding PM-MDTMs, with information provided at induction or in welcome packs. However, the perceived lack of attendance or interest in the meeting by those in management positions potentially sets a tone for the rest of the staff and may remain a barrier to attendance, unless they too are targeted for education regarding the important role for local perinatal review meetings in the clinical governance of the hospital.

The generally low level of accurate description of the meeting and its purpose, particularly among midwifery staff, shows a need for a broader approach to education regarding the importance and benefits of local reviews of perinatal mortality. A general hospital meeting, to which all are regularly invited, may provide an opportunity for this, e.g. Grand Rounds, that is held weekly and facilitated by different groups of staff each week. This would also offer an opportunity to clarify that the meeting is open to all staff and is not invitation-only or for “doctors only.”

As well as improving the education and advertising of the meeting, changing the logistics and modality may improve engagement. As we have seen, the switch to online meetings has increased attendance. Many educational resources have moved online during the pandemic and can be made available for a defined period after their initial airing. This would facilitate engagement with the learning points at a time convenient to staff members, solving both the problems of heavy workloads and requiring meeting logistics to suit all staff. Alternatives which could also help to overcome these barriers and include as many clinical staff as possible in the local review process, may include a regular newsletter detailing learning points from the meeting [5].

### Strengths and limitations

Despite the overall low response rate to the survey, the wide range of HCPs who participated in this study provides a good insight into different knowledge levels and barriers to attendance faced by different groups of staff. Although this study was conducted in a single hospital, many of the barriers faced in attending educational meetings are likely similar to those faced by all health service staff in Irish maternity hospitals. Understaffing is a longstanding crisis across such services both here and in other European countries, and has only been worsened by the COVID-19 pandemic [e.g. 16, 17]. Many staff have worked in other maternity hospitals, allowing insight into different approaches to and perceived benefits of this type of meeting.

Questionnaire-based studies are limited by study design. Free text areas were provided, but not used by all respondents. Limited time and the heavy workload cited by many may have resulted in incomplete or vague answers which were coded as a lack of understanding of the purpose and scope of PM-MDTMs. There was also potential self-selection bias of participants. Nonetheless, this study has provided a valuable insight into both perceptions of and barriers to attendance of PM-MDT meetings. The fact that so many respondents suggested potential improvements suggests an interest and enthusiasm from staff for such reviews and learning opportunities.

## Conclusion

This study has identified the need to improve communication regarding PM-MDTMs, their learning opportunities and benefits in order to improve staff engagement. This in turn should allow us to provide better, more reflective obstetric care. The lack of communication and education regarding PM-MDTMs is likely reflected in the low numbers who could accurately describe the meeting in this study.

Lack of engagement is compounded by significant numbers of new staff, who may be unaware of the existence of the PM-MDTM in the hospital, high clinical workload and low staffing levels, but these issues should not be impossible to overcome. A lack of engagement by senior staff was noted, and this potentially sets the expectation for others’ attendance.

Suggested formats for communication of perinatal mortality reviews are not limited to in-person meetings. The wider availability and increased familiarity with video conferencing software due to the COVID-19 pandemic has provided one avenue to improve engagement in this study. Other alternative methods such as newsletters may be more effective in such contexts where it is difficult to release staff from their duties or where large in-person meetings cannot be facilitated (e.g. currently due to the COVID-19 pandemic). These formats may also help to overcome the issue of finding a meeting time that suits all staff.

## Supplementary Information

Below is the link to the electronic supplementary material.

Supplementary file1 (PDF 229 KB)
