# Supplementary material for: Barriers to attendance at a tertiary hospital’s perinatal mortality meeting
Source: Ir J Med Sci. 2022 Sep 2;192(3):1265–70. doi: 10.1007/s11845-022-03137-0 (PMC9438871; doi:10.1007/s11845-022-03137-0)
Supplement: Supplementary file 1 — Supplementary file1 (PDF 229 KB) [file 11845_2022_3137_MOESM1_ESM.pdf]

Title **Barriers to Attendance at a Tertiary Hospital's Perinatal Mortality Meeting**

Journal **Irish Journal of Medical Science**

Authors **Barbara Burke[1], Sophie Boyd[1], Karen McNamara[1], Keelin O'Donoghue[1,2]**

[1] Pregnancy Loss Research Group, Department of Obstetrics and Gynaecology, Cork University Maternity Hospital, University College Cork, Wilton, Cork, Ireland

[2] The Irish Centre for Maternal and Child Health Research (INFANT), University College Cork, Ireland

Corresponding author – Barbara Burke, bairbre.deburca@gmail.com

Appendix 1 - Questionnaire

Perinatal Mortality MDT Meetings

Thank you for taking the time to answer this survey. The information you give us is confidential and will NOT be used to identify you. We are grateful for your assistance.

This study is about the attendance of staff at Perinatal Mortality MDT Meetings in a University Teaching Hospital, as well as their understanding of its relevance to their work. This survey will help in identifying the reasons for poor attendance as well as providing the most suitable timeframe for staff to attend.

Q 1 What is your current position in the hospital?

- ☐ Consultant
- ☐ Manager
- ☐ Midwife
- ☐ Midwifery management
- ☐ Nurse
- ☐ Neonatal nurse
- ☐ Registrar
- ☐ Researcher
- ☐ Senior House Officer
- ☐ Specialist Midwife
- ☐ Specialist Registrar
- ☐ Other: _______________

Q 2 Where are you currently assigned to work within the hospital?
(please tick all that apply)

- ☐ Antenatal ward
- ☐ Early pregnancy unit
- ☐ Emergency Room
- ☐ Gynaecology /Pregnancy Loss ward
- ☐ Labour ward
- ☐ Neonatal Unit
- ☐ Outpatients department
- ☐ Postnatal wards
- ☐ Theatre complex
- ☐ Ultrasound Department

Q 3 How long have you been working in the Maternity Services?
(O&G, Midwifery, Neonatology)

- ☐ Less than 1 year
- ☐ < 5 years
- ☐ 5 - 10 years
- ☐ >10 years

Q4 How long have you been working in this hospital specifically?

- ☐ Less than 1 year
- ☐ < 5 years
- ☐ 5- 10 years
- ☐ >10 years

Q5 Do you know what the purpose of a Perinatal Mortality MDT meeting is?

☐ Yes ☐ No

How would you explain this meeting to a colleague?

____
____
____

Q6 Do you know what happens at a Perinatal Mortality MDT meeting?

☐ Yes ☐ No

Please outline your understanding of what happens at the meeting;

____
____
____

Q7 Did you know that Perinatal Mortality MDT meetings are held in this hospital?

☐ Yes ☐ No

If 'YES';

a) please state how often they are held

____

b) please state where they are held

____
____

Q8 Have you ever attended a Perinatal Mortality MDT meeting?

☐ Yes – in this hospital
☐ Yes – in another maternity hospital
☐ No

Q8a If Yes – in this hospital;

- **How often have you attended these meetings?**

☐ Occasionally ☐ Sometimes ☐ Regularly

- **Do you find the meetings to be relevant to your clinical practice?**

☐ Yes ☐ No ☐ Sometimes

Please provide feedback of your experience of these meetings;

____

____

____

Q8b If Yes – in another maternity hospital;

- **How often did you attend these meetings?**

☐ Occasionally ☐ Sometimes ☐ Regularly

- **Did you find these meetings to be relevant to your clinical practice?**

☐ Yes ☐ No ☐ Sometimes

Please provide feedback of your experience of these meetings;

____

____

____

Q8c If No, why do you <u>not</u> attend these meetings?

Please give any professional and/or personal reasons;

____

____

____

Q9 Do you think there are particular barriers to staff attending these meetings in this hospital?
If so please state what they are:

____
____
____

Q10 What do you suggest would facilitate improved staff attendance?

____
____
____

Thank you for participating!
